# Advancing universal health coverage in China and Vietnam: lessons for other countries

**DOI:** 10.1186/s12889-020-09925-6

**Published:** 2020-11-25

**Authors:** Wenhui Mao, Yuchen Tang, Tra Tran, Michelle Pender, Phuong Nguyen Khanh, Shenglan Tang

**Affiliations:** 1 Duke Global Health Institute, Duke University, 310 Trent Dr, Durham, NC 27710 USA; 2 Global Health Research Center, Duke Kunshan University, Suzhou, Jiangsu China; 3 Health Strategy and Policy Institute, Hanoi, Vietnam

**Keywords:** Universal health coverage (UHC), Health insurance, Population coverage, Service packages, Financial burden, Vietnam, China

## Abstract

**Background:**

China and Vietnam have made impressive progress towards universal health coverage (UHC) through government-led health insurance reforms. We compared the different pathways used to achieve UHC, to identify the lessons other countries can learn from China and Vietnam.

**Methods:**

This was a mixed method study which included a literature review, in-depth interviews and secondary data analysis. We conducted a literature search in English and Chinese databases, and reviewed policy documents from internal contacts. We conducted semi-structured interviews with 16 policy makers, government bureaucrats, health insurance scholars in China and Vietnam. Secondary data was collected from National Health Statistics Reports, Health Insurance Statistical Reports and National Health Household Surveys carried out in both countries. We used population insurance coverage, insurance policies, reimbursement rates, number of households experiencing catastrophic heath expenditure (CHE) and incidence of impoverishment due to health expenditure (IHE) to measure the World Health Organization’s three dimensions of UHC: population coverage, service coverage, and financial coverage.

**Results:**

China has increased population coverage through strong political commitment and extensive government financial subsidies to expand coverage. Vietnam expanded population coverage gradually, by prioritizing the poor and the near-poor in an incremental way. In China, insurance service packages varied across regions and schemes and were greatly determined by financial contributions, resulting in limited service coverage in less developed areas. Vietnam focused on providing a comprehensive and universal service packages for all enrollees thereby approaching UHC in a more equitable manner. CHE rate decreased in Vietnam but increased in China between 2003 and 2008. While Vietnam has decreased the CHE gap between urban and rural populations, China suffers from persistent disparities among population income levels and geographic location. CHE and CHE rates were still high in lower income groups.

**Conclusion:**

Political commitment, sustainable financial sources and administrative capacity are strong driving factors in achieving UHC through health insurance reform. Health insurance schemes need to consider covering essential health services for all beneficiaries and providing government subsidies for vulnerable populations’ in order to help achieve health for all.

**Supplementary Information:**

The online version contains supplementary material available at 10.1186/s12889-020-09925-6.

## Background

Universal health coverage (UHC) is an ambitious goal wherein all individuals and communities from all countries can access quality health services without suffering financial hardship. UHC has been prioritized in the 2030 Sustainable Development Goals (Target 3.8) and in the World Health Organization’s (WHO) Triple Billion Goals [1, 2]. WHO outlined three dimensions of coverage which can be measured to track a country’s progress towards UHC: population coverage (who is covered); service coverage (which benefits are covered); and financial coverage (what proportion of health service expenses are covered) [3].

Over 100 countries are moving towards UHC with different priorities, and sometimes trade-offs due to limited resources in almost all countries [4, 5]. According to the 2017 WHO and World Bank Global Monitoring Report on UHC, the past decade has seen a rise in service coverage with an increase in catastrophic spending [6]. Service coverage varies across the globe, with a wide variation across East Asia and the lowest coverage in sub-Saharan Africa and Southern Asia [6]. Catastrophic spending from out of pocket payment (OOP) has increased globally, and Latin America, the Caribbean and Asia have the highest rates of OOP [6]. While the availability of services has increased, the financial affordability of healthcare remains an issue of concern. WHO proposed that countries should move towards UHC through healthcare financing reforms [3] which should focus on creating prepaid pooling mechanisms through health insurance schemes, or other health plans to reduce the catastrophic health spending and OOP expenses that push people into poverty. Health insurance reform allows countries to increase access to healthcare by providing financial protection for their population.

As countries move towards UHC, many best practices and lessons can be learned from China and Vietnam. Both countries have made impressive progress towards UHC via government-led health insurance reforms in the past decades. China and Vietnam have both seen remarkable economic development over the last few decades. Since opening up its economy in 1978, gross domestic product (GDP) in China has increased an average of almost 10% a year. China has transitioned into an upper-middle income country and lifted more than 850 million people out of poverty [7]. Vietnam’s economic and political reforms launched under Đổi Mới in 1986, spurred rapid economic growth and helped to transition it into a lower middle-income country, and lifted more than 45 million people out of poverty [8]. The transition from a planned economy to market-oriented mechanisms brought both new opportunities and challenges to the health systems in both countries including a rapidly increasing demand for and expenditure on healthcare. Both countries spent less than 5% of their GDP on Total Health Expenditure (THE) in 2000 (China: 4.47%, Vietnam: 4.84%) which slightly increased in 2017 to above 5% (China: 5.15%, Vietnam: 5.53%). However, only 21.98% of THE came from the Domestic General Government Health Expenditure (GGHE-D) in China while GGHE-D accounted for 34.90% of THE in Vietnam in 2000. GGHE-D increased to 56.67% of THE in China in 2017 and to 45.26% in Vietnam [9].

Despite their similar socio-economic and political histories, China and Vietnam have approached UHC in different ways. China prioritized population coverage and achieved the largest population coverage in history in a relatively short period of time [10, 11]. However, despite this accomplishment, patients continue to face a high financial burden, and insurance only covers a limited number of services [12, 13]. On the other hand, Vietnam’s approach is recognized as a way to make equitable progress towards UHC [14]. Vietnam incrementally extended population coverage to various target groups, especially the poor and vulnerable, and provides them all with a decent service package. Although Vietnam’s health service delivery includes both public-and private systems, it chiefly relies on a vast network of public health care providers from the commune to central level to deliver health services to everyone, but especially those with insurance. As a result, Vietnam has one of the highest scores (73 out of 80) in the WHO’s UHC index on coverage of essential health services [15]. Regardless of different approaches towards UHC, both China and Vietnam have made substantial improvement in population health. Life expectancy in China increased from 66.8 in 1980 to 76.4 in 2017, and the under-five mortality rate decreased from 62.3 to 9.3 per thousand live births during the same period of time [16]. Vietnam almost had the same changes in life expectancy, (67.6 in 1980 and 76.5 in 2017), with a reduction in under-five mortality from 68.3 to 20.9 per thousand live births [16].

Many studies have evaluated the impact of health financing reform and/or presented the lessons learned in different countries. Based on 11 countries experience with UHC, Reich et al. recommended that achieving UHC should be a long-term policy engagement that needs both technical knowledge and political know-how [17]. Lagomarsino et al. provided high-level comparisons among nine Asian and African countries (including Vietnam) in 2015. They found patterns towards UHC included tax revenues to subsidize target populations, small steps that broaden the risk pool and emphasize purchasing services through demand-side financing mechanisms [18]. Tangcharoensathien et al. concluded from their study of Thailand that effective reform should utilize evidence-based practices to develop sound policies [19]. Yip et al. recently summarized the progress and challenges of China’s 10 year health care reform. They found that expanding health insurance coverage for all was fundamental in moving towards UHC and recommended leveraging strategic purchasing, improving primary health-care (PHC) and improving the quality of services for further improvements [20]. Tao et al. identified continued political support and increased health financing to be key lessons from China’s health system reforms [21].

However, limited articles have conducted an in-depth comparison of the design, implementation and impact of the health insurance reforms on UHC. As such, we reviewed the different paths taken by China and Vietnam to achieve UHC including the major initiatives related to their health insurance reforms and analyzed each country’s approach using WHO’s three dimensions. We also identified the important lessons and best practices that other countries can adapt as they move towards UHC.

## Methods

### Study design

We used a mixed methods study to examine China and Vietnam’s approaches to UHC. This included a review of national policy documents, secondary data, case studies, and interviews with key stakeholders. Each country includes two case studies from both a well-developed and moderately developed region - because health insurance policies are determined largely by the economic status of the region [22]. In China, the Eastern province of Jiangsu is an example of a more developed economy, and the Central province of Hubei is used to represent a moderate economy. Since China’s provinces are much larger than those in Vietnam, two prefectures from each province, Zhenjiang in Jiangsu and Jingmen in Hubei, were selected as the primary study area. Vietnam’s locations were chosen for similar economic reasons. Ha Nam is on par with the rest of the country in terms of development, and Bac Ninh province is a more developed region compared to the rest of Vietnam, with 5.86 billion US$ GDP in 2016, it is ranked as the 4th of all provinces in Vietnam. Both provinces are located in the Red River Delta area in north Vietnam.

### Data collection

Data collection was performed between June 2017 and January 2018. This included a literature and document review in English and Chinese using Google Scholar, PubMed, Medline, EMBASE (OVID), Elsevier Science Direct, EBSCO, Web of Science, Wiley, Wan Fang and China Academic Journals (CNKI) database for peer-reviewed articles in Chinese published before December 2017. We also obtained reports and government policy documents from internal contacts.

Data on indicators to measure the three dimensions of UHC (proportion of population insured, insurance reimbursement rates, proportion of households experiencing catastrophic heath expenditure and incidence of impoverishment due to health expenditure) were extracted from reports such as National Health Statistics Report, Health Insurance Statistical Report and National Health Household Surveys carried out in both countries.

In-depth semi-structured interviews were conducted with 16 key stakeholders in China and Vietnam. Interviewees included policy makers, government bureaucrats, and scholars working in the health insurance field. Participants were selected using purpose sampling and snowball sampling. UHC scholars were interviewed in the two countries at the beginning of the study and based on these early interviews, other potential informants were identified and interviewed. In Vietnam, with the help of local researchers from the Health Strategy and Policy Institution, key informants were identified from: 1. Ministry of Health, 2. Vietnam Social Security, 3. Provincial Social Security Bureau and 4. Provincial Health Bureau. In China, key informants were from 1. Provincial Human Resources and Social Security Department (HRSS), 2. Provincial Family Planning Commission (FPC), 3. Municipal Human Resource and Social Security Office, 4. Municipal New Rural Medical Insurance office and 5. District Human Resource and Social Security Office.

The interview instrument included questions about: 1. The history of promoting UHC 2. Changes in essential UHC policies 3. Implementation issues and 4. Current challenges. Interviews were conducted in Chinese in China, and in English in Vietnam. If Vietnamese interviewees had difficulty with English, a local research assistant helped with translation. The interviews were audio recorded and data were extracted from both the interview transcripts and interview notes.

### Data analysis

The analysis of secondary quantitative data focused on: 1. population coverage, 2. reimbursement rates, and 3. financial protection, measured by proportion of households experiencing catastrophic health expenditure (CHE), and incidence of impoverishment due to health expenditure (IHE). Where CHE rate measures the percentage of total households that have paid more than 40% of the household non-subsistence spending OOP, the IHE rate is the percentage of total households that are pushed into poverty due to OOP. The CHE is defined when food expenditure is 40% of total household expenditure.

Qualitative data was transcribed and then analyzed according to themes such as population coverage, financial protection, service coverage, and healthcare financing. Additionally, data on country policy timelines was compiled from analysis of the original policy documents.

## Results

Both countries have steadily increased their Total Health Expenditure (THE) through the introduction of health insurance schemes. When comparing THE as a percentage of GDP, Vietnam currently spends more of its GDP on health. In 2014, Vietnam spent approximately 6% of total GDP on health, while China spent 5.5%. In addition, Vietnam has spent a higher percentage of its government budget on health, when compared to China. Countries shared similar levels of THE in the early 2000’s, however, over the past 15 years, the Vietnamese government increased health expenditure at a higher speed than China.

A summary of major health insurance reforms in China and Vietnam can be found in Additional file 1. In general, China introduced three different insurance schemes successively with different target populations in 1997, 2003 and 2007. Later in 2009, the Health Care System Reform, increased service packages and financial protection, and integrated the New Rural Cooperative Scheme (NCMS) and the Urban Residents’ Basic Medical Insurance (URBMI). In Vietnam, Compulsory Health Insurance (CHI) and Voluntary Health Insurance (VHI) were introduced in 1992. In 2003, the Health Care Fund for the Poor (HCFP) was established. Poorer and older populations were covered incrementally. Law on Health Insurance (LHI) was established in 2009, which rolled the different insurance groups into CHI and aimed to make health insurance compulsory for the whole population in 2014.

### Population coverage

**Fig. 1:**
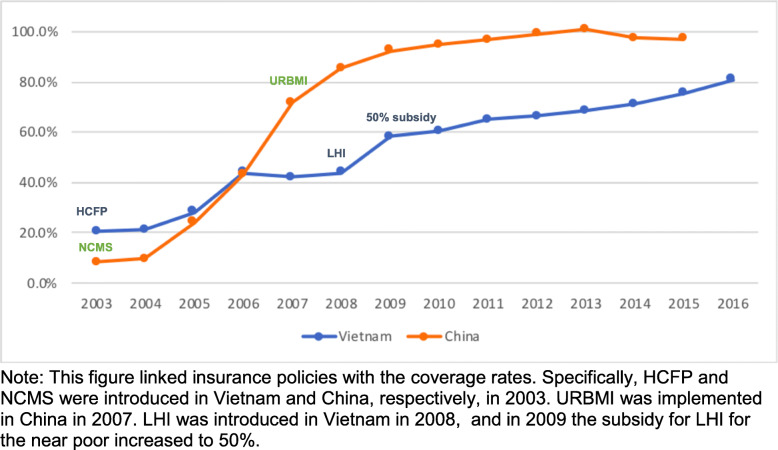
Trends in population coverage by health insurance schemes in China and Vietnam, 2003–2016

The population covered by health insurance has significantly increased in both countries over the past two decades. However, China attained population coverage faster with the implementation of NCMS in 2003 and URBMI in 2007 (Fig. 1 and Table 1). This was achieved by setting low individual premium rates while providing extensive subsidies to attract enrollment. In 2003, the annual premium for NCMS was a flat-rate of CNY30 ($4.6) per enrollee, to which the insured individual, central government, and local government each contributed CNY10 ($1.5). By 2016, the premium reached around CNY570 ($90.1) (CNY120 from the individual, and the remainder from the government). Secondly, local governments under the auspices of the central government led advocacy campaigns to raise awareness of NCMS among all populations.

**Table 1:** Population coverage by insurance group (10,000 population, coverage %)

	Insurance groups	2008		2010		2012		2014	
China	UEBMI	19,996	15.1%	3735	17.7%	26,486	19.6%	28,296	20.7%
	URBMI	11,826	8.9%	19,528	14.6%	27,155	20.1%	31,450	23.0%
	NCMS	81,500	61.4%	83,600	62.4%	80,500	59.5%	73,600	53.8%
	**Total**	132,802	85.4%	133,972	94.7%	135,404	99.2%	136,782	97.5%
Vietnam	Compulsory	3661	43.0%	4698	54.0%	5219	58.8%	5578	61.5%
	Voluntary	313	3.7%	416	4.8%	548	6.2%	756	8.3%
	**Total**	8512	46.7%	8693	58.8%	8882	65.0%	9073	69.8%

Sources: China National Economic and Social Development Statistic Report 2008–2014. China Health Statistics Yearbook 2008–2014; Health Insurance Statistics 2008–2012, 2013–2014, Vietnam Social Security

Vietnam’s approach towards increasing population coverage focused on the poor, vulnerable, and easy to reach populations by first providing government subsidies, e.g. the government is the main payer for Social Health Insurance. In 2014, 45% of SHI members were fully subsidized by the government and 24% were partially subsidized. In addition, in each province HCFP was financed by General government revenue (75%) and Provincial resources (25%) [23]. Secondly various target groups were added into compulsory health insurance programs. There was a steady increase after the implementation of the LHI in 2009 which introduced 50% government subsidies for the near poor. In 2012, the government subsidy was further increased to 70% (Fig. 1 and Table 1).

Table 1 and Fig. 1 illustrate population coverage by different health insurance schemes in the two countries. Of note is that the population covered by NCMS in China declined between 2008 and 2014, which may be attributed to rural to urban migration, as the population covered by URBMI increased over the same time.

Strong political will has been critically important in China and Vietnam. As one Chinese stakeholder mentioned, population coverage became an important performance indicator for local governments which provided strong incentive to promote health insurance coverage. Vietnam by comparison lacked accountability for promoting population coverage at the ministry level. As a VSS officer described, *“[The] VSS is in the government level and at the same level as the MOH. The VSS is also supervised by Ministry of Health, Ministry of Financing, Ministry of Labor, by the Officer of Government and also the Ministry of Internal Affairs regarding human resources and so on. So, there are so many organizations participat [ing] in the management. So, it is not clear who’s responsible for VSS. It’s no one.”*

The Chinese government also channeled financial resources to health insurance. As illustrated by a Zhenjiang stakeholder, *“We have four levels of financial support, the central level, provincial level, city level and also district level support for health insurance fund. … and each year we can get the support on time.”* In contrast, the Vietnamese government failed to provide adequate funding for the HCFP when it was first established. Since the government had trouble financing all medical costs for the poor, the central government couldn’t provide enough financial resources to many less developed provinces. One policymaker from VSS explained, *“This is difficult because the hospitals say ‘ok we can exempt those group from charging the user fee. But the government should pay or fund the health expenditure for these groups’. It [HCFP] fails because the government doesn’t have money.”*

### Policies on service coverage

Service coverage is significantly different between the two countries. In China, coverage is based on the level of funding in different schemes; the scheme with the highest financial support (UEBMI) has the best service coverage and highest reimbursement rates. This results in significant variance in service coverage in China. In Vietnam, service coverage is the same for everyone. The government in 2003 and later 2009, the SHI primarily provides subsidies to cover the poor and older populations, the LHI introduced 50% government subsidies to cover the near poor under SHI. Regardless of the source of financial contributions, all beneficiaries of SHI enjoy the same coverage.

Most health insurance schemes have a list of reimbursable services and drugs [24]. The list for NCMS and URBMI in China is limited due to a low level of funding. The two schemes initially focused on inpatient service coverage, and in 2009, China announced a 50% reimbursement rate for outpatient services. However, as illustrated in Table 2, there is a gap in policy and effective rates of reimbursement due to the implementation of reimbursement lists in different places.

**Table 2:** Reimbursement rates stratified by insurance groups and regions (China)

	Zhenjiang, China	Jingmen, China
Actual Reimbursement Rate		
UEBMI	70%	68%
URRBMI	61%	63%
Reimbursement Rate within policy list		
UEBMI	80%	76%
URRBMI	70%	68%

By contrast, health insurance in Vietnam “is famous for its wide scope even when first initiated” [25]. There is no difference between inpatient and outpatient reimbursement rates. According to a Vietnamese stakeholder, more than 17,000 services and 1530 drugs are covered. By comparison, 4494 services are covered in Zhenjiang, and 3746 in Jinmen. Although Vietnam’s drug list is shorter, the VSS will reimburse 50% of the cost of some other drugs, including those for cancer, if the patient has been covered by insurance continuously for at least 36 months. Co-payment was applied in SHI with a reimbursement rate of 95% for pensioners and the near poor and 80% for the rest of the insured population. Several groups are exempted from this co-payment policy including the poor, children under 6 and merit people. This co-payment policy was the same for in-patients and out-patients. If the patient bypassed the referral system, the VSS would not reimburse for out-patient care and would reduce the payment rate by 40% at the central level, 60% at the provincial level and 70% at the district level for in-patient care.

### Policies and its impacts on financial protection

Table 3 breaks down Catastrophic Health Expenditure (CHE) and Impoverishment due to Health Expenditure (IHE) by insurance, geographic and income quintiles. Considering the major insurance policies in China and Vietnam were both implemented around 2008 (URBMI of China, and the LHI in Vietnam) and the availability of data, we present data on CHE and IHE before and after 2008 for both countries. In 2008, the Chinese CHE rate was 13.5%, while Vietnam’s was 5.5%. Between 2004 and 2014, the CHE rate decreased in Vietnam but China saw an increasing trend of CHE rate during a similar time span. In addition, there is disparity in CHE rates within China and Vietnam. While Vietnam has decreased the CHE gap between urban and rural populations, China has continued to see disparities between populations, stratified by income levels and geographic location. IHE shows a similar pattern. Although the CHE is rather low for the lowest and middle-lower income groups in Vietnam, the IHE rate is still high in these two groups. This implies that poor households still have a high chance of suffering financial hardship when accessing health services.

**Table 3:** Financial protection in China and Vietnam

		Households experiencing catastrophic health expenditure					Household impoverishment due to health expenditure				
		China		Vietnam			China		Vietnam		
		2008	2013	2004	2008	2014	2008	2013	2004	2008	2014
**Insurance status**	Insured	NA^a^		4.4%	5.2%	1.6%	NA^a^		4.1%	3.3%	1.7%
	Uninsured			8.0%	6.9%	2.3%			5.9%	4.5%	2.1%
**By location**	Rural	10.5%	13.0%	6.6%	6.5%	2.6%	3.1%	5.9%	5.2%	4.5%	2.2%
	Urban	16.5%	15.2%	3.1%	3.1%	1.6%	9.7%	7.3%	1.2%	1.1%	0.7%
**By income quintile**	1st(poorest)	19.5%	26.1%	5.5%	7.8%	2.8%	9.3%	11.9%	6.2%	7.5%	1.8%
	2nd	15.5%	15.8%	6.1%	6.0%	2.6%	8.9%	12.0%	12.1%	8.6%	6.4%
	3rd	13.4%	12.4%	6.4%	5.5%	2.0%	7.9%	6.9%	2.2%	1.5%	0.3%
	4th	12.0%	11.5%	5.5%	4.5%	2.0%	4.8%	3.9%	0.2%	0.1%	0.0%
	5th	9.0%	8.1%	4.9%	3.6%	2.0%	2.6%	1.7%	0.0%	0.0%	0.1%
**Total**		**13.5%**	**14.1%**	**5.7%**	**5.5%**	**2.3%**	**6.4%**	**7.0%**	**4.1%**	**3.5%**	**1.7%**

*Sources*: Center for Health Statistics and Information, NHFPC. An Analysis Report of National Health Services Survey in China, 2013 (in Chinese), Health Insurance Statistics 2008–2012, 2013–2014, Vietnam Social Security

^a^IHE and CHE were calculated based on household, while in China, individuals in the same household may have different insurance schemes therefore this indicator is not available in China

## Discussion

Vietnam and China have both demonstrated a strong commitment to achieving UHC and both have made substantial improvements in population health. Health insurance reform over the past two decades has included pooled-funding mechanisms, government subsidies to encourage enrollment, and laws/regulations to promote the implementation of insurance schemes. China established decentralized schemes with a focus on population coverage, but while coverage was high, different locations and schemes offered diverse service packages and financial protection levels. This has led to uneven access to health services and financial protection across the population [12]. Vietnam expanded its compulsory insurance gradually, ensuring wide and unified service coverage for all enrollees. Despite having relatively low population coverage compared to China, Vietnam’s approach seems to have led to financial protection for a higher number of people.

### Population coverage and financial contributions

Both countries provided insurance for easy to reach populations first, with shared financial contributions among individuals, employers and governments, and additional financial support for vulnerable groups. In China, the Ministry of Civil Affairs set up the Medical Financial Assistance Program (MFA) to cover the premiums of NCMS or URBMI and provide extra reimbursement for vulnerable groups. However, with premiums increasing rapidly, we found some provinces failed to increase the MFA subsidies. This indicates the importance of coordination among different sectors in health financing reform. China can teach other positive lessons such as providing strong incentive mechanisms at the local level and using timely allocation of subsidies as an evaluation indicator for local government.

Vietnam’s approach incrementally increased enrollment in compulsory health insurance with priority given to poor populations. The Health Care Fund for the Poor (HCFP) is used to pay premiums for the poor to engage in Social Health Insurance and provides financial support to the poor to cover travel expenses to higher level providers. Vietnam’s approach is suitable for countries with a low proportion of population working in the formal sector. Government subsidies for vulnerable groups help to provide population coverage and prioritize coverage for poor populations. Prioritizing coverage for the poorest population is a recommended approach for countries with limited financial capacity [26]. However, defining and reaching poor populations, can be challenging. Mobilizing the power of local governments and communities could assist in identifying poor populations and improving coverage.

### Service coverage and financial protection

Service coverage between the countries is significantly different and has led to different levels of financial protection. Vietnam provided better and more consistent service coverage for all beneficiaries which promoted accessibility and equity in health services. China successfully covered an astonishing number of people, but the service package was greatly determined by financial contributions. Therefore, service coverage varied across regions and schemes. In addition, due to limited financing, NCMS and URBMI emphasized inpatient services since they are assumed to be the leading cause of catastrophic health expenditure. After several years of implementation there was a significant increase in inpatient services, possibly contributing to China’s increasing cost for services. However, there were also significant increases in health expenditures in Vietnam driven by an increased burden of NCDs and a rapidly aging population. China’s approach demonstrates that while increasing population coverage is incredibly important, providing limited services could lead to increased expenses for the overall health system. In addition, people will use the services that are covered by insurance, so countries should consider cost-effective services for primary care and outpatient services when initially developing their service packages. Differences in service packages have also led to significant differences in access to health services and financial protection between urban/rural and economic groups. Even though the reimbursement policy in China is improving, CHE and IHE remain at a high level, indicating that actual financial burden on households is still high. Vietnam focused on providing a comprehensive and universal service package, increasing coverage slowly, using a pro-poor design in a progressive universalism fashion. Households experiencing CHE or IHE in Vietnam were lower than in China, this has been attributed to the comprehensive service package in Vietnam. Additionally, the service package in Vietnam was the same for all enrollees, (though there are some access issues), overall, Vietnam has approached UHC in a more equitable manner.

Vietnam and China have similar challenges, including ensuring the sustainability of health insurance schemes, and designing an effective cross-subsidization mechanism. To address some of these concerns, Vietnam is working to increase coverage, thereby increasing the financial pooled funding and hopefully decreasing the government’s THE. In addition, China has combined two of their insurance schemes to hopefully reduce administrative costs and increase service coverage. Other countries moving towards UHC should consider designing service packages that cover the essential health services most needed by their population first, and increase population coverage and financial capacity later; using proven cost-effective methods to expand the service packages and increase financial risk protection.

### UHC lessons for other countries

As recommended by WHO, health insurance creates prepaid pooling mechanisms and provides financial protection for service users. In China and Vietnam, the private health insurance market was not well developed and health service providers included a mix of public and private sectors. Countries with similar conditions to China and Vietnam, where health insurance reform has been used as a way to achieve UHC, may need to know that strong political commitment, administrative capacity and committed financial resources are essential to the success of such an endeavor. In addition, they should also establish effective government managed insurance systems. In the initial phase, decentralized insurance designs provide flexibility to adapt the insurance scheme according to different conditions across the country.

Countries moving towards UHC need to address which of the three dimensions of UHC should be prioritized. An important lesson learned when comparing China and Vietnam is that while more of the Chinese population are covered by insurance, access to healthcare services and financial protection are still determined primarily by geographic location and economic status. While in Vietnam, the disparities between economic groups and geographic locations are shrinking. This is primarily due to each country’s priorities – China prioritized population coverage, while Vietnam prioritized service coverage through their pro-poor design. Countries should initially increase government subsidies for health insurance schemes until the pooled mechanism has enough users and providers enrolled. While this will increase health spending initially, as more people enter the pooling mechanism, the less the government will need to subsidize.

### Limitations

The effective rates of reimbursement for the Vietnam provinces were not included in this study, and we were unable to find data that could reflect access to “essential” health services. The CHE and IHE were not available for both countries in the same years, and the income quintiles represented different absolute living standards in China and Vietnam and shouldn’t be compared with each other directly. In addition, this study did not analyze the quality of health services in China and Vietnam and we did not obtain information from patients or service providers.

## Conclusion

Political commitment, sustainable financial resources and administrative capacity are strong driving factors in achieving UHC through health insurance reform. When designing health insurance systems, prioritizing population coverage, service coverage and financial protection, consistent service coverage for essential health services and government subsidies for vulnerable populations are encouraged to entitle everyone has the right to health.

## Supplementary Information

**Additional file 1.** Major initiatives of health insurance reforms.

## Data Availability

Data and information collected for this study is available upon request, please contact shenglan.tang@duke.edu.

## Abbreviations

CHE: Catastrophic heath expenditure
CHI: Compulsory Health Insurance
GGHE-D: Domestic General Government Health Expenditure
GDP: Gross domestic product
HCFP: Health Care Fund for the Poor
IHE: Incidence of impoverishment due to health expenditure
NCMS: New Rural Cooperative Scheme
OOP: Out of pocket payment
LHI: Law on Health Insurance
MFA: Medical Financial Assistance Program
MHRSS: Ministry of Labor and Social Security
SHI: Social Health Insurance
THE: Total Health Expenditure
UHC: Universal Health Coverage
UEBMI: Urban Employees' Basic Medical Insurance
URBMI: Urban Residents’ Basic Medical Insurance
URRBMI: Urban and Rural Residents’ Basic Medical Insurance
VSS: Vietnam Social Security
VHI: Voluntary Health Insurance
WHO: World Health Organization
