# Supplementary material for: Advancing universal health coverage in China and Vietnam: lessons for other countries
Source: BMC Public Health. 2020 Nov 25;20:1791. doi: 10.1186/s12889-020-09925-6 (PMC7690086; doi:10.1186/s12889-020-09925-6)
Supplement: Supplementary file 1 — Additional file 1. Major initiatives of health insurance reforms. [file 12889_2020_9925_MOESM1_ESM.docx]

**Additional file 1 Major initiatives of health insurance reforms**

| **China** | | **Vietnam** | |
| --- | --- | --- | --- |
| Year | Initiatives and key activities | Year | Initiatives and key activities |
| 1997  2003  2007  2009  2009-2011  2012-2015  2016 - Present | Urban Employee Basic Medical Insurance (UEBMI) led by the Ministry of Labor and Social Security (MHRSS) to cover government and formal sector employees  State Council establishes New Cooperative Medical Scheme (NCMS) to cover rural residents  Urban Resident’s Basic Medical Insurance (URBMI) to cover children, students, and informal sector laborers in urban areas  Health Care System Reform  Stage 1 included:   - Increasing population coverage to 90% by expanding URBMI - Increasing financial resources by increasing premium contributions - Increasing financial aid to the poor for participating in insurance schemes   Stage 2 included:   - Increasing policy reimbursement rate to 75% for inpatient services and 50% for outpatient services - Removing deductible and maximum payment - Increasing reimbursement rate for the poor and disabled - Establishing a supplementary insurance program to cover diseases with catastrophic health expenditure   Stage 3 included:   - Integrating NCMS and URBMI into one scheme led by the MHRSS known as the Urban and Rural Residents’ Basic Medical Insurance (URRBMI) | 1992  1994  1998  2002  2003  2005  2007  2009  2014 | Led by the Ministry of Health, Compulsory Health Insurance (CHI) to cover formal sector workers, pensioners, and the disabled and Voluntary Health Insurance (VHI) to cover students, children, dependents of CHI enrollees, the self-employed and farmers  Free exemption policy for those who have rendered meritorious national service, and the poor  All health insurance schemes merged into one  Free health services established for population aged over 100 years  The Health Care Fund for the Poor (HCFP). HCFP is used to pay for premium of the poor to engage in Social Health Insurance and provides financial support the poor to cover travel cost to higher level of provider.  Free health care for children under the age of 6  Government subsidy extended to population aged 85 and over  Law on Health Insurance (LHI) rolling the different insurance groups into CHI to cover workers in the formal sectors, pensioners and those of national merit, the poor and the near poor, children under the age of 6 and students  LHI revised to make health insurance compulsory for whole population |

Source: author summarized from policy documents.
